# Diagnostic utility of α-methylacyl CoA racemase (P504S) & HMWCK in morphologically difficult prostate cancer

**DOI:** 10.1186/1746-1596-5-83

**Published:** 2010-12-22

**Authors:** K Kumaresan, Nandita Kakkar, Alka Verma, Arup Kumar Mandal, Shrawan Kumar Singh, Kusum Joshi

**Affiliations:** 1 Department of Histopathology, Post Graduate Institute of Medical Education and Research, Sector 12, Chandigarh, 160012, India; 2 Department of Urology, Post Graduate Institute of Medical Education and Research, Sector 12, Chandigarh,160012, India

## Abstract

**Background:**

To evaluate the diagnostic utility of alpha-methylacyl CoA racemase (P504S) & HMWCK (34beta E12) in morphologically difficult prostate cancer.

**Methods:**

A total of 1034 cases were reviewed and divided into benign (585) malignant (399) and suspicious (50). Immunohistochemistry with HMWCK and AMACR was done on the 50 suspicious cases along with controls.

**Results:**

Forty nine suspicious cases were resolved by using both markers where as 1 case was resolved by further support with CD68. The original diagnosis was changed in 15 of 50 (30%) suspicious cases from benign to malignant, one case from benign to high grade PIN and in one case from malignant to benign. Change of diagnosis was seen in 17 of 50 (34%) suspicious cases with a significant p value of 0.002. The overall diagnosis was changed in 17 of 1034 cases (1.64%) of prostatic disease (p < 0.001).

**Conclusions:**

A combination of HMWCK and AMACR is of great value in combating the morphologically suspicious cases and significantly increasing the diagnostic accuracy in prostate cancer. Although, in this study the sensitivity and specificity of HMWCK and AMACR were high, yet it should be used with caution, keeping in mind all their pitfalls and limitations.

## Introduction

The diagnosis of prostatic cancer (PC) is based on a combination of architectural, cytological and ancillary features rather than any single diagnostic feature none of which is absolutely sensitive and specific. Accurate tissue diagnosis can be very challenging due to the presence of either a small focus of cancer or due to the presence of many benign mimickers of malignancy like adenosis, sclerosing adenosis, atrophy, partial atrophy, basal cell hyperplasia, clear cell cribriform hyperplasia, post atrophic hyperplasia, nephrogenic adenoma, mesonephric hyperplasia, radiation atypia, seminal vesicle and cowpers glands [1-3]. Due to the widespread use of serum PSA as a mass screening test for prostate cancer there has been an ever increasing number of prostate needle biopsies and hence the need to give an accurate diagnosis despite the limitations. Approximately 40-50% of patients with limited cancer had moderately advanced or advanced carcinoma on final radical prostatectomy [4]. Therefore, underdiagnosis of a small focus of prostatic adenocarcinoma might delay early treatment and cause severe adverse consequences for patients. Benign glands contain basal cells, which are absent in cancerous glands and hence the use of basal cell markers (HMWCK 34βE12, p63, CK5/6) to label the basal cells when faced with an ambiguous lesion [5-7]. More recently a positive marker for prostate carcinoma, α-methylacyl CoA racemase (AMACR) has been reported to have sensitivity ranging from 82-100% [8,9]. So, the aim of this study was to use immunohistochemistry in morphologically suspicious prostate and to assess how a combination of HMWCK (34βE12) and AMACR (p504S) contributes to a final diagnosis. In addition, the sensitivity and specificity of HMWCK as well as AMACR for the detection of prostate cancer and benign prostatic tissue were also evaluated.

## Materials and methods

In this study all prostate biopsies/transurethral resection of prostate (TURP) specimens/prostatectomies from 2005 to 2008 were retrieved from the archives of the Dept of Histopathology, PGIMER, Chandigarh, India. These were 1082 in number and of these 48 cases were excluded from the study due to inadequate material or because of the biopsy being nonrepresentative. Finally, 1034 cases were selected for further evaluation. These included 575 needle biopsies, 414 TURP specimens, 21 simple prostatectomies, 18 radical prostatectomies and 6 cystoprostatectomies. The hematoxylin and eosin stained slides of all cases were reviewed by three histopathologists and divided into 3 categories-benign (585 cases), cases with suspicious foci (50 cases) and malignant (399 cases). The cases with suspicious foci were further subjected to immunohistochemistry (IHC) with HMWCK (34βE12) and AMACR (p504S). To act as control 16 cases of benign prostatic tissue (tumorous/nontumorous) and 25 cases of frank adenocarcinoma of various grades were selected and subjected to IHC with the same markers.

The 50 suspicious cases consisted of 25 needle biopsies, 19 TURP specimens, 4 simple prostatectomies and 2 radical prostatectomies. The age ranged from 48 to 85 years. PSA was available in 19/50 cases and ranged from 7 to 100. Of these, 30 cases were given a histopathology report of being benign of which 14 were labeled as benign prostatic hyperplasia (BPH), 14 as no evidence of malignancy (NEM) and 2 as inflammation (INFL). Of the rest, 19 cases were reported as prostatic carcinoma (PC) and 1 case as high grade prostatic intraepithelial neoplasia (HGPIN). These 50 suspicious cases were further labelled into three categories (Cat). **Category 1**- Initial pathology diagnosis given at the time of routine reporting; **Category 2- **Reviewed (by three histopathologists) diagnosis before IHC; **Category 3**- Final diagnosis after IHC with both HMWCK and AMACR. Based on light microscopy, the 50 suspicious cases in category 2 were further classified into three subcategories A, B and C. **Category 2A **was labelled as atypical small acinar proliferations (ASAP) and consisted of 40 cases exhibiting small crowded glands showing some, but not all of the architectural and cytological features of adenocarcinoma. These glands lacked significant cytologic abnormality, occupied < 5% of the biopsy area or raised the possibility of one of the mimics of prostate carcinoma. In two cases however the glands were larger but crowded. **Category 2B **consisted of 7 cases of frank prostatic carcinoma with co-existent atypical foci (PC+ATF). In 3 cases the differential diagnosis was between cribriform PIN and cribriform carcinoma, in 2 cases the marked focus was suspicious for HGPIN and in 2 cases the marked focus looked morphologically different (more benign) from the co-existing carcinoma. These foci were picked up for evaluation for the purpose of learning in case such foci were encountered independently in needle biopsies without the co-existing carcinoma. **Category 2C **consisted of 3 cases of camouflaged morphology (CM)-They were camouflaged by crush artifacts and inflammation and needed IHC for a categorical diagnosis. One case showed extensive crush artifact with suspicious glands, the second case showed dense chronic inflammation along with suspicious glands and the third case showed dense lymphoplasmacytic inflammation with atypical cells.

### Immunohistochemistry for HMWCK and AMACR

The blocks from all suspicious and control cases were cut and mounted on poly l- lysine coated glass slides. Endogenous peroxidase activity was blocked by freshly prepared 0.3% hydrogen peroxide in methanol for 20 minutes. Subsequently, heat-induced epitope retrieval was performed by using citrate buffer at PH 6. IHC was performed by using a rabbit monoclonal anti-AMACR antibody (p504 S, clone no 13H4 1:50 dilution) and a monoclonal anti-HMWCK antibody (clone no 34βE12 1:50 dilution).

### Evaluation of immunohistochemistry: Criteria of positive/negative staining

#### AMACR

Positive staining pertains to dark diffuse or granular, cytoplasmic or luminal, but circumferential. The percentage positivity was graded from 0+ to 3+ as follows:- 0% cells (0+, negative), 1-10% cells (1+, mild), 11-50% cells (2+, moderate), > 51% cells (3+, strong). The adjacent benign glands should not show more than weak, partial (noncircumferential) staining if any. Negative staining pertains to no staining or focal, weak noncircumferential fine granular staining.

#### HMWCK

The basal cell marker, HMWCK was interpreted as negative/positive and continuous/discontinuous.

### Statistical analysis

Data was analyzed using the statistical package SPSS version 16.0 for MS-Windows (SPSS Inc., Chicago, IL).The significance of HMWCK and AMACR immunostains in resolving the atypical foci was analyzed using chi square test. Significance was assumed at a p value less than 0.05.

## Results

### Expression of HMWCK/AMACR in controls

#### Benign controls

In all the 16 benign controls (Table 1), HMWCK highlighted the presence of basal cells in the form of moderate to strong cytoplasmic continuous positivity. In 14 (87.5%) of the 16 benign controls IHC for AMACR was totally negative. However, in 2 cases there was focal, weak and noncircumferential luminal staining observed in benign glands which were interpreted as negative. In seminal vesicle (3 cases) the HMWCK stained the basal cells and the AMACR was negative.

**Table 1:** Expression of HMWCK/AMACR in 16 benign controls

Antibody	Benign prostates -16	Seminal vesicle-3
HMWCK	Positive-moderate to strong, cytoplasmic, continuous	Positive
**AMACR**	14 cases-negative 2 cases-focal, weak, non circumferential-Non specific	Negative

#### Malignant controls

**Figure 1:**
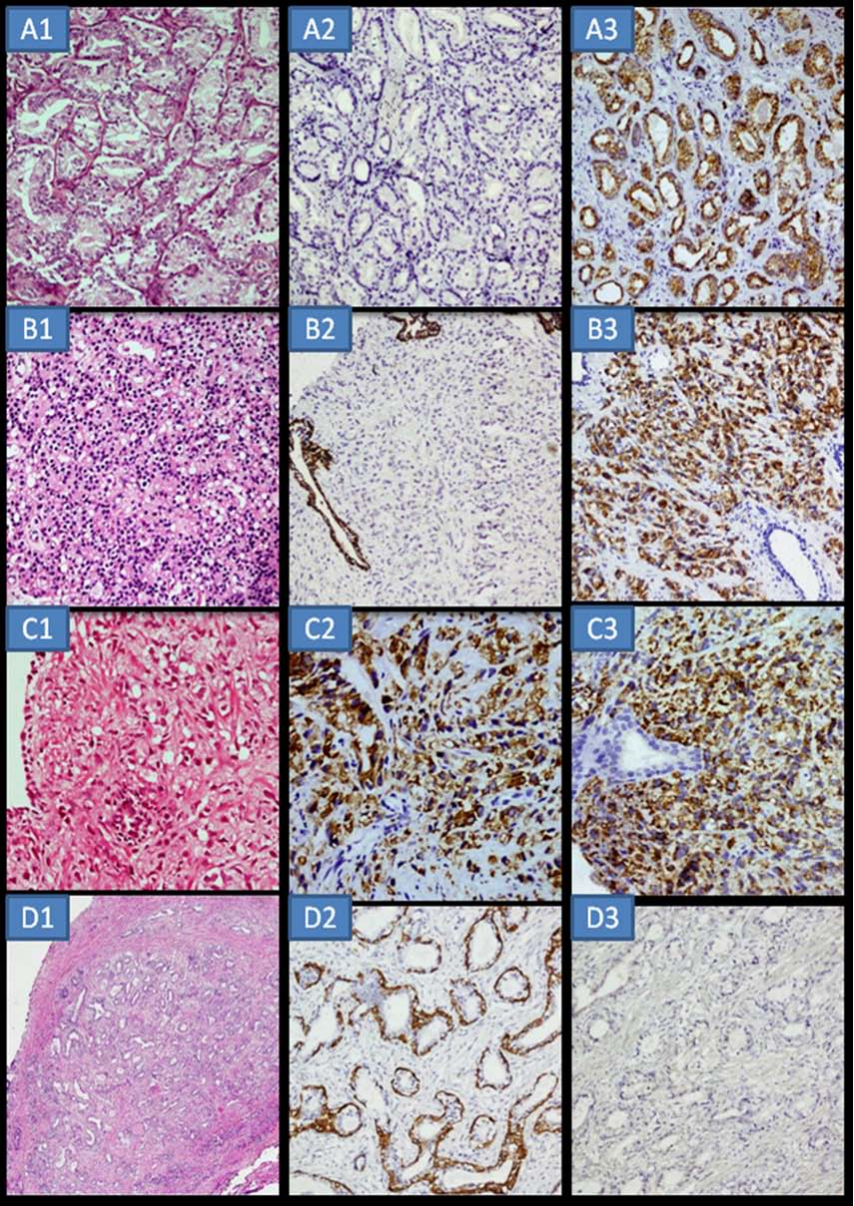
**A1-H&E Low grade prostate carcinoma x400**. A2-HMWCK negative staining in the malignant glands × 400. A3-AMACR strong 3+ luminal circumferential positivity in the malignant glands × 400. B1- H&E High grade prostate carcinoma × 400. B2- HMWCK negative in the malignant cells × 400. B3- AMACR strong 3+ positivity in the malignant cells × 400. C1-H&E High grade prostate carcinoma × 400. C2- HMWCK strongly positive in the malignant cells × 400. C3- AMACR strong 3+ positivity in the malignant cells × 400. D1-H&E Focus of ASAP-adenosis × 200. D2- HMWCK strongly positive in the basal cells × 400. D3- AMACR shows negative staining × 400.

Of the 25 cases of malignant controls (Table 2), 8 cases had associated HGPIN in the adjacent area. In 21/25 malignant control the HMWCK was negative in the malignant glands. However in 4 cases i.e.16% HMWCK was strongly positive in the malignant cells/glands. AMACR was positive in 23/25 malignant cases and negative in two cases with the majority i.e 20/25 (80%) showing moderate to strong positivity (Figure 1A and 1B). The location of staining was usually luminal to subluminal and circumferential, but some cases showed diffuse cytoplasmic staining or mixture of both pattern. All the 8 cases with HGPIN showed strong continuous to occasionally discontinuous positivity for HMWCK and moderate to strong positivity for AMACR. As highlighted in the Table 2, two high grade (Figure 1C), one intermediate and one low grade prostatic carcinomas were positive for both markers. So, in this study we concluded that AMACR has a sensitivity of 92% and a specificity of 100% whereas HMWCK has a sensitivity of 100% and a specificity of 84%.

**Table 2:** Expression of HMWCK/AMACR in 25 malignant controls

Antibody	Low grade-10	Intermediate-9	High-6	HGPIN-8
**HMWCK**	9-negative	8-negative	4-negative	8- Positive
	1-positive	1-positive	2-positive	Continuous/discontinuous
**AMACR**	9-positive	All positive	5 positive	8- positive-
	1-negative		1 negative	moderate to strong

#### Expression of HMWCK and AMACR in 50 suspicious cases

**Figure 2:**
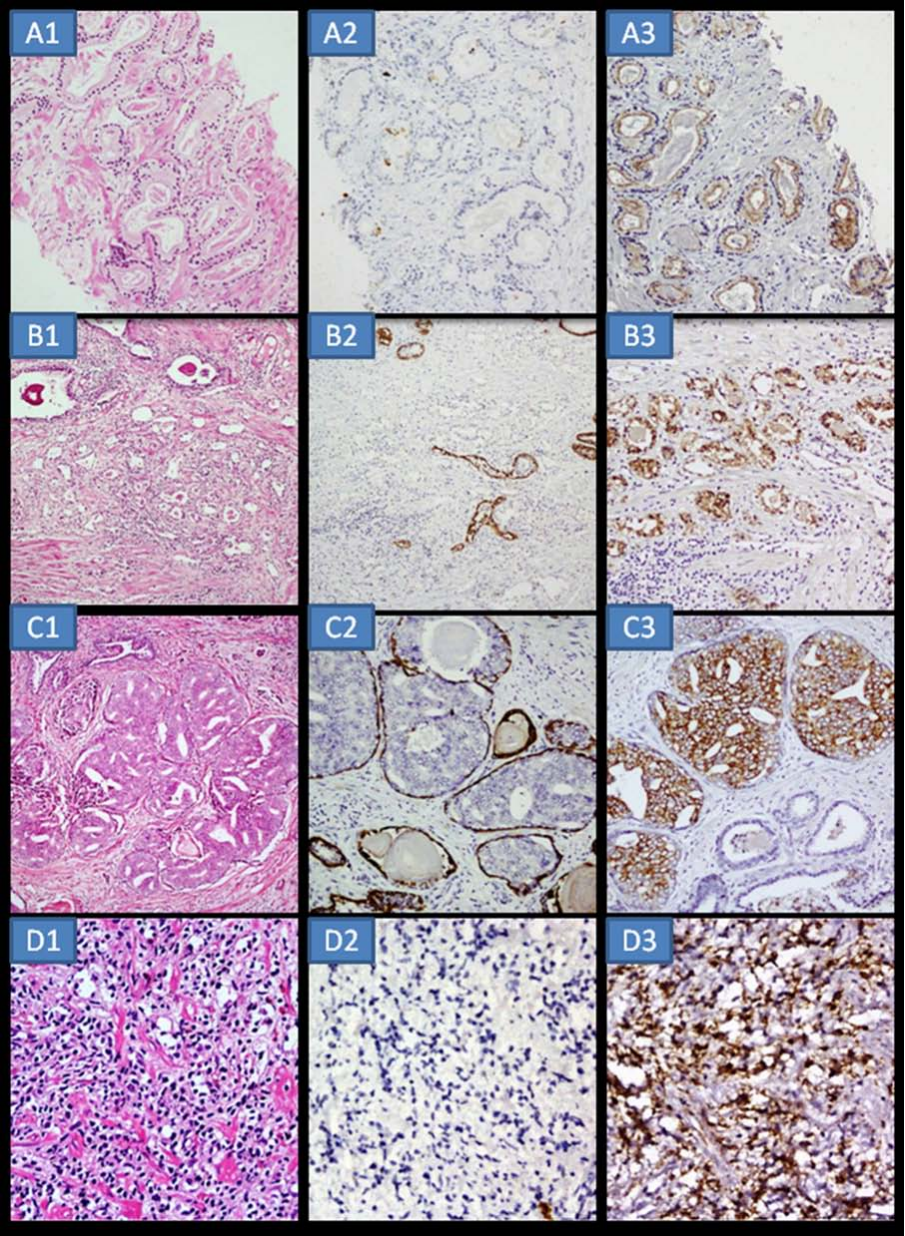
**A1-H&E ASAP focus in NBX-PC × 400**. A2- HMWCK shows negative staining × 400. A3-AMACR strong 3+ luminal circumferential positivity × 400. B1-H&E ASAP focus in TURP- PC × 400. B2- HMWCK negative staining × 400. B3-AMACR strong 3+ luminal circumferential positivity × 400. C1-H&E Atypical focus-HGPIN × 400. C2- HMWCK strongly positive in the basal cells × 400. C3- AMACR strong 3+ positivity in the cells × 400. D1-H&E Camouflouged morphology- Inflammation × 400. D2-AMACR negative staining in the atypical cells × 400. D3- CD 68 strong positivity in the atypical cells × 400.

Taking into consideration the morphology, clinical details and staining with HMWCK and AMACR the 50 suspicious cases (Table 3) were recategorized. All ASAP's showing moderate to strong circumferential granular or diffuse positivity with AMACR and a negative staining with HMWCK were relabeled as adenocarcinoma. Those with a negative AMACR and positive labeling with HMWCK in the basal cells as adenosis, atrophy and those with positive labeling with HMWCK in the luminal cells as basal cell hyperplasia. Suspicious foci with larger glands exhibiting positive staining with both AMACR and HMWCK as PIN. In **category 2A**, 14/40 ASAP cases were initially diagnosed as benign prostatic hyperplasia (Cat 1) and of these 5 were relabeled (Cat 3) as carcinoma (Figure 2A and 2B), 2 as carcinoma with HGPIN, 3 as basal cell hyperplasia and 4 as adenosis. Of the 14/40 ASAP cases initially diagnosed as no evidence of malignancy (Cat 1), 5 were relabeled (Cat 3) as carcinoma, 1 as carcinoma with HGPIN, 1 case as HGPIN, 4 as adenosis (Figure 1D) and 3 as atrophy. Of the 10/40 ASAP (Cat 2A) cases initially diagnosed as carcinoma (Cat 1) remained so. One case of ASAP (Cat 2A) initially diagnosed as inflammation (Cat 1) was relabeled as carcinoma (Cat 3) and one case initially diagnosed as HGPIN, remained so. In **category 2B **there were 7 cases of frank prostatic carcinoma with atypical foci which looked different from the co-existing carcinoma. Five atypical foci were labeled as HGPIN of which 3 were labeled as cribriform HGPIN (Figure 2C) and one each as adenosis and basal cell hyperplasia.. **In category 2C**, one case (initially reported as carcinoma) with crush artifact showed negativity for HMWCK and moderate positivity for AMACR hence confirming the diagnosis of prostate carcinoma. The second case (initially reported as inflammation) with inflammation and squamous metaplasia, the suspicious large glands showed negative HMWCK staining and strong positivity (3+) for AMACR, hence relabeled as carcinoma. The third case (initially reported as malignancy) with dense inflammation admixed with atypical cells showed negativity for AMACR. Subsequently immunostain for CD68 was performed which highlighted these atypical cells as histiocytes giving a diagnosis of nonspecific inflammation (Figure 2D).

**Table 3:** Correlation between original (Cat 1), reviewed (Cat 2) and final diagnosis (Cat 3)

Orig diag Cat 1	Rev diag Cat 2-A,B,C	Final diagnosis after IHC- Cat 3								
		PC	PC+ HGPIN	PC+ ADEN	PC+BCH	BCH	ADEN	ATROPHY	HGPIN	SUSP
BPH-14	**ASAP-14**	**5**	**2**	0	0	3	4	0	0	0
NEM-14	**ASAP-14**	**5**	**1**	0	0	0	4	3	**1**	0
INFL-2	**ASAP-1**	**1**	0	0	0	0	0	0	0	0
	**CM-1**	0	**1**	0	0	0	0	0	0	0
PC-19	**ASAP-10**	7	3	0	0	0	0	0	0	0
	**CM-2**	1	0	0	0	0	0	0	0	**1**
	**PC+ATF-7**	0	5	1	1	0	0	0	0	0
HGPIN-1	**ASAP-1**	0	0	0	0	0	0	0	1	0

Cat-category, Orig-original; Rev-reviewed; diag-diagnosis, BPH- benign prostatic hyperplasia; PC-prostatic carcinoma; HGPIN- high grade prostatic intraepithelial neoplasia; NEM-no evidence of malignancy; INFL- inflammation; BCH- basal cell hyperplasia; ADEN- adenosis; CM -camouflaged morphology; ASAP-atypical small acinar proliferation, ATF- atypical foci, Susp- suspicious

#### Change of diagnosis after immunohistochemistry-

In 17/50 i.e. 34% of the suspicious cases there was a change of diagnosis based on morphology, clinical details and staining with HMWCK and AMACR. In 15/50 i.e. 30% the diagnosis was changed from benign to malignant, in one from benign to HGPIN and in one from malignant to benign. This change was statistically significant with p value of < 0.002. Of the total 1034 cases, the change of diagnoses was seen in 17/1034 cases i.e 1.64%. This change in diagnosis too was statistically significant with p value < 0.001. Of these 17 cases there were 12 needle biopsies and all twelve were done for a clinical suspicion of prostatic carcinoma. PSA levels were available in 9/12 cases only, and were high in all cases ranging from 7 to 100 ng/ml. Ten of these cases had foci of ASAP which on morphology were very suspicious. In all needle biopsies the percentage of tumor found was 5-15% and was of Gleason score 3+3 ie 6. In 5 cases TURP was done with a clinical diagnosis of benign prostatic hyperplasia. PSA was available in only one case and was high (10 ng/ml). The TURP specimens revealed a very small focus of malignancy ie 3- 5% with a Gleason score of 3+3 = 6. In 15 cases with change of diagnosis from benign to malignant the HMWCK was negative and AMACR was positive in all cases with 2+ in 4 cases and 3+ in 11 cases.

## Discussion

Prostate carcinoma is the most common form of cancer in men and the second leading cause of death. The advent of prostate-specific antigen screening has led to a significant increase both in the number of prostate needle biopsies performed and in the number of difficult biopsies with a small foci of adenocarcinoma and atypical glands suggestive but not diagnostic of adenocarcinoma. The diagnosis of prostate cancer is made by use of traditional histological parameters, including architecture, nuclear features and ancillary features (if necessary) rather than any single diagnostic feature [1]. Tissue diagnosis of prostate cancer can be difficult due to the presence of either a small focus of cancer or due to the many benign mimickers of malignancy like adenosis, atrophy, partial atrophy, basal cell hyperplasia, clear cell hyperplasia, post atrophic hyperplasia, nephrogenic adenoma, mesonephric hyperplasia, radiation atypia, seminal vesicle and Cowpers glands [1,2]. Prostatic biopsies occasionally contain proliferative foci of small atypical acini that display some but not all features diagnostic of adenocarcinoma. Such foci have been described by a wide variety of terms which are synonymous like suspicious, atypical focus, and atypical small acinar proliferation (ASAP) suspicious but not diagnostic of malignancy. The most accepted terminology however is ASAP. Needle biopsies signed out as ASAP include such lesions as HGPIN, benign mimickers of cancer (atypical adenomatous hyperplasia, BCH, atrophy), reactive atypia and many cases that in retrospect show minute carcinoma but contain insufficient cytological or architectural atypia to establish a definitive diagnosis of cancer. The likelihood of prostate cancer on subsequent biopsy in men with a diagnosis of ASAP on initial biopsy is 21% to 49% [10,11].

Amongst the mimickers, atrophy [2] and partial atrophy [12] are frequently misdiagnosed as prostate carcinoma. Low power maintenance of lobular architecture at least in part, uniform cytology, absence of prominent nucleoli and presence of a basal cell layer [2] takes you away from a diagnosis of carcinoma. Partial atrophy has a lobular to disorganized and diffuse pattern growth with glandular crowding which are paler and angulated and the nuclei are occasionally larger with more prominent nucleoli. The presence of patchy/absent basal cells and pseudo nerve invasion makes it mimic an adenocarcinoma. Adenosis is defined as a focus of very crowded small glands suspicious for cancer, admixed with more recognizably benign glands. Glands have pale to clear cytoplasm with nuclei showing a lack of a very prominent nucleoli [13,14] and possess a fragmented or continuous basal cell layer [2]. Basal cell hyperplasia is a mimicker of prostate carcinoma especially in needle biopsies. It is usually characterized by a nodular expansion of uniform round glands associated with a cellular stroma. Morphologically there are residual small lumina lined by secretary cells with clear cytoplasm and these are surrounded by multiple layers of basal cells which are dark with a scant cytoplasm and a round or oval spindled hyperchromatic nuclei [2] and which stain with the basal cell markers. Nephrogenic adenoma is a benign metaplastic response of the urothelium to injury and can rarely affect the prostatic urethra. Extension of small tubules of nephrogenic adenoma into the underlying prostatic fibromuscular stroma can lead to a misdiagnosis of low grade prostatic adenocarcinoma. Presence of Cowpers glands in TURP specimens may resemble both low grade adenocarcinoma or foamy gland carcinoma both of which can have a bland cytology. Occasionally seminal vesicles sampled on needle biopsy can also be a source of over diagnosing prostatic adenocarcinoma. A common finding on needle biopsy is the dilated irregular lumen of the seminal vesicle seen at the edge of the tissue core with surrounding clusters of small glands.

In recent years basal cell markers and prostate biomarker Alpha-Methylacyl- CoA- Racemase (AMACR) have been used as adjuvant to morphology in diagnostically challenging cases with a very high sensitivity and specificity. This has increased the diagnostic accuracy of prostate cancer worldwide. Basal cell markers such as HMWCK (34βE12) and CK 5/6 and P63 are very useful for demonstration of basal cells as their presence argues against a diagnosis of invasive prostatic carcinoma [7]. There are several caveats associated with the use of basal cell markers for the diagnosis of PC. The first is that one is relying on a negative finding ie lack of basal cell staining to make a positive diagnosis of carcinoma. The second caveat is that 5% to 23% of benign prostatic gland [8,14], up to 50% of cases of adenosis in a fragmented pattern with some totally negative glands [15], 23% cases of atrophy [16], 44%-75% cases of nephrogenic adenoma [17] and 66% cases of mesonephric hyperplasia may lack basal cell staining and thus a negative basal cell marker immunostain alone does not lead us towards a diagnosis of malignancy. Seminal vesicle and ejaculatory duct epithelium is usually positive for basal cell markers where as data regarding Cowper's glands is contradictory [18]. Hence, one must be cautious in interpreting negative basal immunostains as they are supportive of a diagnosis of PC in the appropriate H&E context. The final caveat is the fact that there are reports of PC positive for basal cell markers [14,19]. Most of these positive cases are high grade PC's and are usually readily diagnosed based on H&E appearance. But there are reports of gland forming invasive acinar adenocarcinomas reported to harbor basal cells in 1% of cases [19]. Some of these could represent carcinoma outpouchings from HGPIN glands or alternatively flat HGPIN. Thus, although basal cell markers are an extremely useful adjunct, it is important to recognize that the diagnosis of cancer is based on the absence of a detectable positive basal cell layer. Therefore, a sensitive and specific positive immunohistochemical marker is necessary to increase the level of confidence in establishing a definitive diagnosis of malignancy in prostate pathology.

AMACR also known as racemase or p504 S is an enzyme recently identified by cDNA subtraction and microarray technology. It is a sensitive and specific IHC marker found to be consistently up regulated in prostate carcinoma [20]. A notable advantage to an AMACR immunostain is that a diagnosis of malignancy is substantiated by a positive signal rather than loss of a signal. Multiple studies have now evaluated the utility of AMACR immunostain in the diagnosis of PC. But there are varied reports regarding the expression of AMACR in prostate cancer which ranges from 62% to 100% [8,9,20-24]. Some morphological variants of prostatic carcinoma that pose a particular diagnostic problem and for which immunohistochemistry is particularly needed to establish the diagnosis of malignancy have been reported to express less AMACR immunoreactivity compared with their more conventional counterparts. Zhou et al [22] found AMACR expression in 70-77% of pseudohyperpalstic carcinomas and 62-68% of foamy gland cancers. In addition to prostate cancer, AMACR positivity has been demonstrated in 90% cases of HGPIN suggesting that the possibility of HGPIN must be carefully excluded by morphology and the use of basal cell markers, before AMACR positivity is used to establish the diagnosis of adenocarcinoma. AMACR positivity in HGPIN has been found to vary from weak to strong. AMACR expression is also identified in 4%-21% of benign prostatic glands [20,21] in up to 18- 27% of cases of adenosis [25] and in 18-58% of cases of nephrogenic adenoma [26,27]. Although AMACR is a useful immunohistochemical marker for prostate cancer, it has significant limitations. It is so emphasized that AMACR should be interpreted in the appropriate morphological context and in conjunction with basal cell markers.

On reviewing 1034 prostate cases, 50 ie 4.8% atypical cases were deleniated. These were divided into three categories (40 cases of ASAP, 7 cases of prostate cancer with atypical foci and 3 cases of camouflaged morphology) and subjected to further analysis by immunohistochemistry with HMWCK and AMACR. In various studies in literature the incidence of atypical biopsies ranged from 0.4-23% with a mean of 5.5% [28]. In the index study based on morphology, clinical details and the interpretation of the two markers we were able to resolve 49 of the 50 atypical cases (98%) and by addition of CD68 immunostain the remaining case was also resolved. Jiang et al [29] found that the AMACR and HMWCK (34βE12) immunohistochemistry in the workup of 41 foci of so-called atypical small acinar proliferation (ASAP) led to a 76% agreement rate between the 3 pathologists participating in the study. Zhou et al [22] demonstrated that, of 115 prostate biopsies diagnosed as atypical by an expert pathologist, 34 (30%) were changed to a final diagnosis of cancer based on a positive AMACR immunostain. Browne et al [30] also found that the use of a cocktail of both a basal cell antibody and an AMACR immunostain helped resolve the diagnosis in 70% (86/123) of ''challenging'' prostate needle biopsies. Sanderson et al [31] used p63/AMACR cocktail to reclassify 2 (29%) of 7 atypical prostate needle biopsies as prostatic carcinoma. Molinie et al [32] were able to resolve 89% of 104 "ASAP" in needle biopsies using a p63/AMACR antibody cocktail compared with only 53% with CK 5/6. Kunju et al [23] were able to resolve 27 (93%) of 29 atypical biopsies after immunostaining with AMACR and basal cell markers.

In the present study, taking into consideration the morphology, clinical details and in conjunction with IHC with HMWCK and AMACR, 24/40 ASAP (Cat 2A) cases with a negative staining with HMWCK and moderate to strong positive staining with AMACR were finally categorized as prostatic carcinoma of which only 10 were initially (Cat1) reported as carcinoma. Two cases showing strong positive staining for AMACR and also strongly highlighting the basal cell layer with 34βE12 were diagnosed as HGPIN (Cat 1- one reported as HGPIN and one as NEM), In conjunction with morphology, a negative AMACR stain and a positive basal cell layer with 34βE12, 8 cases were labeled as adenosis (Cat 1 reported as BPH and NEM), 3 as basal cell hyperplasia (Cat 1 reported as BPH) and 3 cases as atrophy one of which being partial atrophy (Cat 1 reported as NEM). These 14 cases finally labeled as adenosis, atrophy and basal cell hyperplasia were however strongly positive for 34βE12 and negative for AMACR and none of these were interpreted as PC initially (Cat 1). Hence in the ASAP category 2A there was a change in diagnosis in 15 of the 40 cases. In 14 cases it was from benign to malignant and in one case it was from benign to HGPIN. There were 7 cases of frank PC with atypical foci. These foci were picked up for evaluation for the purpose of learning in case such foci were encountered independently in needle biopsies without the carcinoma. Of these 5/7 were finally categorized as HGPIN and one case each as adenosis and basal cell hyperplasia. Three cases initially labeled as cribriform carcinoma was now labeled as cribriform PIN and this brought down the grade of the tumor from intermediate to low grade. The last category was labeled as camouflaged morphology (3 cases) in which there was inflammation and crush artifacts. These were finally categorized as PC in 2 cases (Cat 1-one case reported as inflammation and other as PC) and histiocyte rich inflammation in one case (Cat 1 reported as PC). Thus in this study the initial diagnosis made on routine reporting was changed in 17/50 ie in 34% of the atypical cases. In 15/50 i.e. 30% the diagnosis was changed from benign to malignant, in 2% (1/50) from benign to HGPIN and in 2% (1/50) from malignant to benign. The change of diagnosis in these 17 patients was communicated to the treating urologists for further follow up and action. The case in which the diagnosis was changed from malignant to benign, in the mean time had had a radical prostatectomy done and there was dense inflammation and no tumor in the whole specimen. Another case where the diagnosis was changed from benign to malignant on a needle biopsy underwent a radical prostatectomy and there was tumor (Gleason score 3+3 = 6) present in both the lobes.

The most common reason for the diagnostic error in this study was that the malignant foci were very limited (3-10%) and there are well known reasons for the difficulty in diagnosing limited prostatic adenocarcinoma. Firstly the limited number of cancerous glands may only be a few acini available for histopathological evaluation. AMACR has now been demonstrated to be a highly sensitive prostatic cancer marker (sensitivity 80-100%) for small focal prostatic carcinomas [32-34]. Secondly there is no single feature specific and sufficient for the diagnosis of prostate cancer. The diagnosis is based on a combination of architectural and cytological features and the presence of extracellular material such as blue tinged secretions or crystalloids^,^. Almost any of these histological diagnostic features can be occasionally seen in benign conditions of the prostate [11]. Thirdly, the consequences associated with a false positive or negative diagnosis can be very serious like unnecessary prostatectomy, radiation exposure or a delay in effective treatment. In this study 94% cases with change of diagnosis were from benign to malignant/premalignant that is cases were being underdiagnosed at our institute due to the presence of limited adenocarcinoma. It would be better if the diagnosis of suspicious but not diagnostic of malignancy is given to such cases. The other reasons for the error were inflammation, crush artifacts, missing out on HGPIN (not looking at high power judiciously), and diagnostic errors with benign mimics of carcinoma. In the five TURP specimens the single chip with the small focus of carcinoma was missed on routine reporting. In one case the only chip with carcinoma was lying outside the cover slip- a technical error.

Among the control cases AMACR was positive in 23/25 PC and negative in 14/16 benign controls with focal weak noncircumferential positivity in 2 cases (considered as negative). HMWCK was positive in all 16 benign controls and in 4/24 PC cases. So, in this study we concluded that AMACR has a sensitivity of 92% and a specificity of 100% whereas HMWCK has a sensitivity of 100% and a specificity of 84%. Four cases of malignant controls (two high grade, one intermediate grade and one low grade) had positivity for both HMWCK and AMACR immunostaining. Rarely high grade prostate carcinomas can express HMWCK and this is usually not a diagnostic problem as AMACR is positive in the malignant cells (as was in our cases) and morphology is diagnostic of malignancy [7]. Most of the positive cases reported in literature [14] are high grade PC's but there are reports of gland forming invasive acinar adenocarcinomas reported to harbor basal cells in 1% of cases [19]. Staining in a basal cell pattern can be explained by the possibility of retention of basal cells by early invasive carcinoma or that some glands seem to be outpouchings of HGPIN or alternatively flat HGPIN. This false positive staining is seen more with HMWCK than with p63. Some antigen retrieval methods have been implicated in nonspecific staining of prostate cancer cells by basal cell markers [29,35]. It was noted that the hot plate antigen retrieval method though better for the overall staining, caused nonspecific immunoreactivity in the tumor cells. The pepsin predigestion and microwave retrieval methods did not cause this phenomenon, however some benign acinar basal cells failed to stain with these methods.

So, we conclude that in conjunction with morphology and clinical scenario, a combination of HMWCK and AMACR is of great value in combating the morphologically suspicious cases and thus significantly increasing the diagnostic accuracy in prostate cancer. However, the limitations of both the markers should be kept in mind.

## Abbreviations

AMACR: α-methylacyl CoA racemase; ASAP: atypical small acinar proliferation; ADEN: adenosis; ATF: atypical foci; BCH: basal cell hyperplasia; BPH: benign prostatic hyperplasia; CAT: category; CM: camouflaged morphology; HGPIN: high grade prostatic intraepithelial neoplasia; HMWCK: high molecular weight cytokeratin; INFL: inflammation; NEM: no evidence of malignancy; PC: prostate carcinoma; TURP: transurethral resection prostate.

## Competing interests

The authors declare that they have no competing interests.

## Authors' contributions

KK, NK and KJ participated in selecting cases, interpretation of results and writing of the manuscript. AK and KK carried out the immunohistochemistry. SKS and AKM provided the clinical details of the patients. All authors read and approved the final manuscript.
